# P21 Lupus or else?

**DOI:** 10.1093/rap/rkad070.042

**Published:** 2023-09-27

**Authors:** Faidra Laskou, James Carroll, Ernest Wong

**Affiliations:** Queen Alexandra Hospital, Portsmouth, United Kingdom; Queen Alexandra Hospital, Portsmouth, United Kingdom; Queen Alexandra Hospital, Portsmouth, United Kingdom

## Abstract

**Introduction:**

Systemic Lupus Erythematosus (SLE) is a systemic, autoimmune inflammatory disease with an unknown aetiology and various presentations. Wide spectrum of clinical manifestations can be seen in different infectious diseases, which may mimic SLE. Syphilis is a disease caused by the spirochete Treponema pallidum. Secondary syphilis is characterised by fever, lymphadenopathy, malaise, weight loss and rash but hepatitis, tenosynovitis, polyarthritis, and renal impairment have also been reported. Syphilis mimicking SLE is widely reported; it is though not part of routine infectious screening. We describe a case of secondary Syphilis mimicking SLE in a 47-year-old female, presented with skin and joint manifestations.

**Case description:**

A 47-year-old female, presented with 5 weeks history of polyarthralgia, rash, affecting the torso, face and malaise. No significant past medical history reported. She presented with a diffuse slightly urticated indurated plaques over the face, scalp, neck, back, abdomen and axillary/upper arm regions, with some minimal surface scale. A suspicion of a connective tissue disease (CTD) was raised; a skin biopsy was taken. Hydroxychloroquine (HCQ) 200mg BD and prednisolone 40mg OD were initiated by dermatology. Migratory arthritis (wrists, elbows, knees) was reported. Her CRP was 99mg/L, ESR 55 mm/hr, with ferritin at 274 ug/L. She has mildly deranged LFTs (ALT of 55 U/L, ALP 498 U/L). Considering an autoimmune aetiology, and in view of the uncertainty of diagnosis her rheumatoid screen, anti-double-stranded DNA antibodies (anti-dsDNA) and lupus anticoagulant were requested and were negative. Complement levels (C3/C4) were normal. HIV, hepatitis, EBV and CMV screen was negative. Anti-nuclear antibodies (ANA) were weakly positive with a reported fine speckled pattern while her cardiolipin IgM and IgG were mildly raised. Her antineutrophil cytoplasmic antibodies (ANCA) were reported as weakly atypical positive with marginally raised MPO of 9U/ml and negative PR3 levels. The significance of those positive results at this stage were unclear in view of the patient presentation. She was then treated for suspected SLE and methotrexate (MTX) was introduced. Two weeks later, she developed right posterior uveitis, mouth ulcers, alopecia and worsening polyarthralgia. In view of deterioration toxoplasma, syphilis serology and a CXR were done as the skin biopsy at this point was suggestive of sarcoid. Syphilis serology was in keeping with active syphilis infection (RPR and confirmation test positive). Her HCQ and MTX were stopped and benzathine penicillin G was given. The final diagnosis was of secondary syphilis with granulomatous rash, arthritis, uveitis, and non-scarring alopecia.

**Discussion:**

This was a very interesting case that involved at least 4 specialties before a final diagnosis was made. Malaise and arthritis have several causes and the presence of a rash can be explained by other CTDs,vasculitides, drug eruptions and infections. Our patient did not present with the “typical” syphilitic rash involving the palms and soles. This patient's constellation of signs and symptoms could be fully compatible with both SLE and secondary syphilis; however, only weakly positive ANA levels with negative dsDNA, normal complements levels make the diagnosis of SLE questionable; our patient did not fulfil the SLE (SLICC) or the EULAR/ACR classification criteria as only the presence of synovitis, weakly positive ANA levels and weakly positive cardiolipin antibodies (medium or high titres are required for diagnosis) were present. Furthermore, the skin changes were not fully compatible with SLE, hence a skin biopsy was performed in view of the diagnostic uncertainty.Uveitis, on the other hand, has been described in patients with SLE but it is usually affecting the anterior uveal. Posterior uveitis has been described in SLE but it is less common compared to anterior uveitis. In syphilis, posterior uveitis and pan uveitis are the most common ocular presentations; It is arguable that the presence of posterior uveitis in our patient could have also raised the suspicion of an alternative diagnosis.Retrospectively, and following further discussion with the patient, she reported that one of herpartners, developed similar symptoms few weeks ago and he was referred to GUM for further evaluation. Having this information earlier, would have potentially guided us to look for all STD’s before further immunosuppression is given. This case highlighted the importance of recognising atypical features in patients who are referred with suspected CTD’s so appropriate investigations can be carried out before significant immunosuppression is given.

**Key learning points:**

It is key to exclude infective causes in suspected CTD cases with diagnostic uncertainty and atypical features

Important to include the dermatology team and consideration of a skin biopsy when the main organ involved is the skin

Ensure sexual health history is taken from all new patients that present with symptoms compatible of connective tissue or inflammatory diseases

When patients do not respond to treatment as expected, as in our case, ensure other causes have been excluded before escalating treatment and consider a multidisciplinary approach when there is diagnostic uncertainty

In a skin biopsy showing granolumatous inflammation, and in cases of uveitis, consider syphilis among the differential diagnosis

Consider syphilis in routine screening as it remains the great imitator; often mimics features of acute and chronic inflammatory disorders

